# Impact of variable economic conditions on the cost of energy and the economic viability of floating photovoltaics

**DOI:** 10.1016/j.heliyon.2024.e32354

**Published:** 2024-06-04

**Authors:** Leonardo Micheli, Fredy A. Sepúlveda-Vélez, Diego L. Talavera

**Affiliations:** a Dept. of Astronautical, Electrical and Energy Engineering (DIAEE), Sapienza University of Rome, Rome, 00184, Italy; b Dept. of Electronic and Automatic Engineering, University of Jaén, Las Lagunillas Campus, Jaén, 23071, Spain; c Advances in Photovoltaic Technology (AdPVTech), University of Jaén, Las Lagunillas Campus, Jaén, 23071, Spain

**Keywords:** Floating photovoltaics, Economic analysis, Cost analysis, Sensitivity analysis, LCOE, NPV

## Abstract

This work evaluates the effects of economic conditions’ variations on the costs and viability of floating photovoltaics, a novel solution where modules are installed on or above water. A sensitivity analysis of key economic criteria is conducted across multiple European countries, first generating country-specific baseline scenarios and then introducing systematic variations into the input parameters. The results show that capital expenditure and electricity prices, which have both experienced significant variations in recent years, have the largest influence on the net present value and the internal rate of return. Similarly, capital expenditure and discount rate are found to be the most influencing factors for the levelized cost of electricity. Overall, this study contributes to the literature by identifying the correlations between the economic variables and the viability of floating photovoltaics. The findings can be used to assess the effectiveness of potential government policies and support mechanisms and to evaluate the viability of this technology under varying national and international economic conditions.

## Highlights

• The viability of floating photovoltaics is assessed for variable economic conditions.
• A sensitivity analysis of three economic criteria is presented for 25 countries.
• The capital expenditure is the dominant factor for determining the net present value.
• Some mechanisms to support the deployment of floating photovoltaics are proposed.
• More detailed results are provided for five representative countries.

## 1 Introduction

Photovoltaic (PV) modules can directly convert solar irradiance into electricity without a working fluid or moving parts. Driven by an unprecedented drop in costs and by the need for a larger renewable energy share, PV is expected to become the most installed energy technology worldwide by 2027 [1]. However, the massive deployment of PV modules will require the conversion of a significant amount of land, which might threaten biodiversity [2] and compete with essential activities such as agriculture [3].

Floating photovoltaics (FPV) consists of modules installed on water basins [4] and is one of the solutions proposed to alleviate land competition. The economic feasibility of this technology has already been investigated in the literature [5], as discussed in Section 2. However, the techno-economic analyses presented so far have typically offered valuable snapshots of the current viability of FPV, but have neglected the dynamic nature of economic variables and PV costs. Economic conditions can indeed undergo significant changes [6,7], making the conclusions of analyses based on fixed input values unreliable. Nonetheless, only a few works have investigated the correlations between economic conditions and FPV viability. Specifically, Rizvi et al. [8] evaluated the economics of an FPV site in India and found that the net present value (NPV) and the levelized cost of electricity (LCOE) were proportional to the percentage increase in PV module price. In addition, Boduch et al. [9] investigated the correlations between investor's equity, auction price, and NPV of an FPV system in Poland. While important, these analyses only considered variations in a limited number of parameters, ignoring potential fluctuations in other factors, such as electricity prices. Also, these works focused on single sites, making it difficult to generalize the results.

Hence, there is a need for a large-scale analysis of the influence of key economic factors on FPV viability. For this reason, this work presents a comprehensive sensitivity analysis of the economics of FPV. Multiple European countries are considered to account for a diverse range of weather and economic conditions. The analysis begins by establishing a realistic reference scenario for each country using actual country-specific figures to examine three economic assessment criteria: the NPV, the Internal Rate of Return (IRR), and the LCOE. Subsequently, a sensitivity analysis is conducted to demonstrate the influence of the various parameters on these three criteria.

The present work contributes to the development and deployment of FPV by investigating, on an unprecedented scale, the correlations between the economic viability of FPV and economic variables, whose variability has often been neglected in previous techno-economic studies. Additionally, it assesses the robustness of these correlations across a variety of climates and conditions, addressing a gap in previous sensitivity analyses, which focused on individual FPV sites and a limited number of parameters. The results of this analysis can inform institutions and prospective owners/investors about the economic profitability and cost-competitiveness of FPV, considering possible variations in key techno-economic factors. Specifically, these findings enable the evaluation of how government policies, support mechanisms, technology development, and national and international economic conditions may affect the economic feasibility of FPV systems.

The paper is structured as follows. Section 2 provides an overview of the most common criteria for the economic assessment of PV projects and reviews the techno-economic studies on FPV, explaining the motivation behind the present work. In Section 3, the methodology and the main techno-economic parameters are described. Finally, Section 4 presents and discusses the results.

## 2 Background and motivation

In the first part, this section describes the criteria commonly used to analyze the cost competitiveness and profitability of PV installations, discussing their advantages and disadvantages. The second part presents a review of the economics of floating photovoltaics, critically analyzing current knowledge on the topic, and explaining the needs that the present work aims to address.

### 2.1 Criteria for economic assessment of PV projects

Various criteria can be used for the economic evaluation of PV systems. These include the net present value (NPV), the internal rate of return (IRR), the profitability index (PI), the benefit-to-cost (B/C) ratio, the discounted payback time (DPBT), and the levelized cost of electricity (LCOE) [10,11].

As also shown in the subsequent subsection (2.2), NPV and IRR are the most common criteria for evaluating the profitability of PV systems [[12], [13], [14], [15]]. The NPV expresses the difference between the present worth of the (in and out) cash flows generated throughout the lifetime of the PV system [10]. The IRR is the discount rate that makes the NPV equal to zero. In particular, an investment should be accepted when the NPV is positive and sufficiently large, and when the IRR is greater than the minimum acceptable rate of return (hurdle rate), which represents the opportunity cost of capital to the investor. The NPV explicitly determines the magnitude of the profits, while the IRR is used to accept/reject a project as it allows a quick comparison with the hurdle rate [16].

Other criteria, such as PI and B/C, may be used to compare and rank potential investment opportunities. Specifically, the PI is a criterion based on the NPV and can be used to differentiate projects that have the same NPV. The criterion B/C offers similar results to PI [10]. However, they are not as commonly found in PV studies as the NPV and the IRR.

An additional parameter that can be employed is the DPBT, which expresses the number of years needed for the cumulative discounted cash flow to equal the initial investment. However, even if easy to understand, the DPBT offers only a partial overview of the economic viability of a project, as it does not consider the cash flows produced after the initial investment is recovered. Hence, it might hide sound financial opportunities for those deciding to invest in a PV system. Simple payback time (SPBT) is not recommended either, as it does not take into account the time value of the money. Indeed, the use of SPBT implies that there are no opportunity costs to the investor (i.e., the investor's discount rate is assumed to be zero) [17].

The LCOE, on the other hand, does not directly measure the profitability but expresses the cost of producing electricity from a given source. It is well-known in energy studies as it allows comparing diverse technologies, even when there are differences in scales of operation, investment sizes, and/or operating periods. For instance, the LCOE is commonly used to compare the cost of electricity generated by renewable and conventional energy resources [10]. Additionally, the LCOE can be employed to assess the cost competitiveness of a technology in the energy market. Technologies whose costs are higher than the electricity price are unlikely to be included in the national electricity mix [18,19].

Because of the aforementioned reasons, this study assesses the economics of FPV under a variety of scenarios by analyzing the two most common profitability criteria (NPV and IRR) as well as the LCOE. The LCOE indeed facilitates comparisons of cost competitiveness between FPV and other energy technologies and also helps understand its economic viability within each of the investigated national electricity markets.

### 2.2 Economic status of FPV

Despite being in a relatively early development stage, the techno-economic viability of FPV has been investigated on several occasions. Specifically, most studies on this topic have conducted comparative analyses to assess the cost competitiveness of this technology compared to land-based photovoltaics (LPV). Niccolai et al. [5] have recently compiled a comprehensive and critical review of the technical, economic, and environmental aspects of FPV and specifically discussed the LCOE values reported in the literature. They concluded that, typically, the LCOE for FPV is still higher compared to LPV, because of the higher FPV installation costs. For instance, Boduch et al. [9] carried out a technical and economic assessment of a 1 MWp FPV system in Poland and found that, even in the best-case scenario, FPV cannot currently economically compete with LPV. Indeed, because of the lack of economy of scale and of a fully developed supply chain, the costs related to the installation of FPV are still higher than in LPV [20].

However, contrasting results are available in the literature on the economics of FPV. For example, the results of a techno-economic feasibility study conducted by Goswami et al. [21] showed that the LCOE of FPV in West Bengal, India, is already lower than that of LPV. Similarly, Islam et al. [22] found that an FPV system installed in Dhaka, Bangladesh, would achieve a lower LCOE than land-based installations. A different study estimated similar payback times for ground-mounted and floating PV systems in a semi-arid region of Brazil [23]. Semeskandeh et al. [24] conducted a techno-economic-environmental feasibility study of a 5-kW FPV and found 22 % shorter DPBTs compared to LPV, thanks to the cooling effect of water. Makhija et al. [25] assessed the viability of an LPV system, an FPV system, and a grid extension for a remote village in India using the System Advisor Model (SAM) software developed by the National Renewable Energy Laboratory (NREL), USA. They concluded that the FPV system was the most feasible solution according to various criteria (i.e., NPV, IRR, cost of energy, environment cleanliness, and social acceptance). Hafeez et al. [26] also used SAM to conduct a techno-economic analysis of an urban lake site in Pakistan. They found FPV significantly more economically viable than LPV, thanks to the elimination of the land costs, which minimized the NPV and DPBT. Ocampo et al. [27] evaluated the techno-economic feasibility of an FPV system in the Philippines and found LCOE lower than the market price, positive NPV, and favorable IRR.

While most techno-economic comparative analyses have focused on individual systems, larger-scale investigations have also been conducted in some cases. For example, Cromratie Clemons et al. [28] assessed the economic and environmental performance of FPV in Thailand compared to various types of LPV technologies. They found lower LCOE and DPBT and higher NPV and IRR for FPV compared to LPV. Oliveira-Pinto et al. [29] analyzed the economic feasibility of FPV technologies based on a thorough and up-to-date market research. The study, conducted in Brazil, Spain, and the U.K., reported LCOEs ranging from 5.03 €_cents_/kWh to 9.62 €_cents_/kWh, in all cases above those of LPV systems. Micheli et al. [30] found that, in Europe, FPV could already be cost-competitive with traditional LPV only in conditions of high tilt angles and of improved water-induced cooling.

Additional studies have investigated the economics and cost-competitiveness of FPV in absolute terms, without a direct comparison with LPV or other energy sources. For instance, Jawad et al. [31] assessed the potential contribution of small-scale FPV systems to the achievement of the U.N. Sustainable Development Goal 7 in Bangladesh, considering the technical, economic, environmental, and social impacts of FPV. Through the analysis of several economic criteria, such as NPV, IRR, PBT, and LCOE, the authors concluded that FPV would be a financially viable solution for a case study in the capital city. Islam et al. [32] investigated the techno-economic feasibility of a 10 MW floating solar PV system in Malaysia and found an LCOE of USD 0.052/kWh and a payback period of 9.5 years. Faruqui et al. [33] conducted a technical potential and economic feasibility assessment for selected water bodies in Bangladesh using SAM. Through the analysis of NPV, IRR, and LCOE, they found that all the investigated projects were profitable. Baptista et al. [34] evaluated the FPV potential in Portugal. The authors found a high 14-year DPBT, despite the promising NPV and IRR values. Micheli and Talavera [19] assessed the cost competitiveness and the profitability of FPV in Europe, finding lower-than-electricity-price LCOEs in the southernmost countries.

Previous studies offer valuable insights into the current economics of FPV. However, global and local economic mechanisms, along with the development of the FPV market, can lead to significant variations in the economic conditions, potentially affecting the findings of those analyses. For instance, the average total installed costs of photovoltaics have dropped by 80 % since 2010, contributing to a nearly 90 % decrease in LCOE [6]. Additionally, European electricity prices have experienced significant fluctuations in recent years, with severe repercussions on the profitability of PV [7]. For example, the COVID-19 outbreak and the related lockdown measures lowered the electricity demand and consequently the prices in several countries [9]. Subsequently, the energy crisis that followed reversed this trend, causing the prices to rise to unprecedented levels between 2021 and 2022 [7]. Nonetheless, the impact of these potential variations on FPV economics remains still relatively unexplored. In their aforementioned work, Boduch et al. [9] investigated how changes in investor's equity and auction price could affect the NPV of an FPV system in Poland. In addition, Rizvi et al. [8] analyzed the effect of a rise in PV module prices on the economics of an FPV-grid integrated system in Chhattisgarh, India. However, both works reported results for individual locations and a limited number of parameters.

Therefore, a systematic study of the repercussions of changes in economic conditions on the FPV cost competitiveness and profitability is lacking. Specifically, there is a need to understand how changes in economic conditions affect the FPV cost competitiveness and profitability. This is the aim of the current work, which establishes baseline scenarios for each country (based on current conditions) and then introduces systematic variations in input parameters to assess their impact on each of the investigated criteria. Compared to previous sensitivity analyses [8,9], conducted at individual locations, the study considers 25 European countries and multiple parameters and criteria, so that the effects of each variable can be assessed in different weather and economic conditions.

## 3 Methodology

### 3.1 Economic and cost analysis

As discussed in the previous section, the present work analyzes the profitability using the Net Present Value (NPV), and the Internal Rate of Return (IRR). Additionally, the cost of electricity generated by FPV is expressed through the Levelized Cost of Electricity (LCOE). The equations employed for the calculation of these parameters are presented in this subsection and are based on the methodologies employed in previous works [19,35].

The *NPV* of a PV system is the difference between the present worth of the cash flows (in and out) generated throughout the lifetime of the PV system [10]. This is given by the expression:

(1) $$NPV=-CAPEX+PW\left[CI\left(N\right)\right]-PW\left[{FPV}_{OM}\left(N\right)\right]+PW\left[DEP\;\left(N_{d}\right)\right]$$

where CAPEX is the capital expenditure (i.e., the initial investment cost) of the FPV system, *PW[CI(N)]* is the present worth of the cash incomes over the lifetime (*N*, expressed in years) of the FPV system, *PW[PV*_*OM*_*(N)]* refers to the present worth of its operation and maintenance expenditure, *PW[DEP(N*_*d*_*)]* is the present worth of the tax depreciation during the depreciation period (*N*_*d*_).

The present worth of the cash income over the lifetime is calculated as [35]:

(2) $$PW\left[CI\left(N\right)\right]={p∙Y}_{F}∙\sum_{n=1}^{N}\frac{{\left(1-r_{d}\right)}^{n}}{{\left(1+d\right)}^{n}}{∙\left(1+r_{p}\right)}^{n}∙\left(1-T\right)$$

where *p* is the electricity price, *Y*_*F*_ is the energy yield of the FPV system, *r*_*d*_ is the annual system degradation rate*, d* is the discount rate, *r*_*p*_ is the rate at which the electricity price varies every year, and *T* is the applicable income tax rate.

The lifetime operation and maintenance costs are calculated as:

(3) $$PW\left[FPV_{OM}\left(N\right)\right]=\sum_{n=1}^{N}\frac{OMEX∙\left(1-T\right){∙\left(1+r_{om}\right)}^{n}}{{\left(1+d\right)}^{n}}$$

where *OMEX* is the yearly operation and maintenance expenditure and *r*_*om*_ the rate at which it varies every year.

Last, the present worth of tax depreciation term is calculated from the annual tax depreciation (*D*_*n*_) as:

(4) $$PW\left[DEP\left(N_{d}\right)\right]=\sum_{n=1}^{N_{d}}\frac{D_{n}}{{\left(1+d\right)}^{n}}∙T$$

The IRR criterion can be defined as the discount rate (*d*) that makes the NPV equation equal to zero:

(5) $$0=-CAPEX+PW\left[CI\left(N\right)\right]-PW\left[{FPV}_{OM}\left(N\right)\right]+PW\left[DEP\;\left(N_{d}\right)\right]$$

As aforementioned, from an economic point of view, an investment in an FPV system is acceptable if a) IRR exceeds a profitability threshold fixed by the future investor/owner and b) IRR is greater than the organization's weighted average cost of capital (WACC), at least. WACC is the cost that the owner or investor of the PV system must pay to finance the initial investment. In this work, the WACC is calculated according to Ref. [36].

The cost analysis is conducted through the Levelized Cost of Electricity (LCOE), which quantifies the cost of producing each kWh of electricity over a PV system lifetime (in €/kWh). In this paper, it has been calculated according to the equation below:

(6) $$LCOE=\frac{CAPEX+PW\left[FPV_{OM}\left(N\right)\right]-PW\left[DEP\left(N_{d}\right)\right]}{Y_{F}∙\sum_{n=1}^{N}\frac{{\left(1-r_{d}\right)}^{n}}{{\left(1+d\right)}^{n}}}$$

This criterion enables the comparison of the costs of different energy technologies. Moreover, as described earlier, it makes it possible to evaluate the cost-competitiveness of a source within the national electricity market [19]. Specifically, the LCOE can be compared with the electricity price (p) and, if LCOE < p, one can conclude that the energy source could likely be included in the energy mix. Indeed, in European electricity markets, the energy mix is made of electricity offered at prices equal to or lower than the national electricity price. Therefore, since the LCOE expresses the cost of producing electricity from a given source, if LCOE > p, the energy could unlikely be offered at competitive prices (i.e. at prices lower than the electricity price). On the other hand, if LCOE < p, the energy could likely be offered at prices lower than the national electricity prices and therefore be included in the energy mix.

### 3.2 Input parameters

A review of the typical values for the techno-economic parameters employed in the current economic estimation is presented below. The economic values presented in this review were sourced from a previous study [19] and are reported in Table 1. These were employed to generate the base case scenario for each investigated country.

**Table 1:** *Economic parameters used in this study, sourced from* Ref. [19].

Country	Day ahead average price (2010–2021) [€_(2021)_/MWh] [[37], [38], [39], [40], [41], [42], [43], [44], [45], [46], [47], [48], [49], [50], [51], [52], [53], [54]]	Avg. nominal lending interest rate in 2010–20 [i_l,_ %] [55,56]	Avg. Inflation in 2010–2021 [i, %] [57]	Nominal equity IRR in 1900–2010 [d_s,_ %] [58]	Corporate Tax Rate [T, %] [59]	WACC [%]
Albania	65.8	8.5	2.1	15.8	15.0	10.4
Austria	46.0	4.5	1.9	8.7	25.0	5.6
Belarus	67.6	13.6	17.8	20.2	18.0	14.3
Belgium	50.4	4.1	1.7	6.9	25.0	4.6
Bosnia and Herz.	65.8	5.6	0.7	15.0	10.0	8.9
Bulgaria	52.7	7.4	1.8	8.5	10.0	7.3
Cyprus	60.8	5.1	0.6	11.5	12.5	7.2
Croatia	66.3	9.5	1.1	15.0	18.0	10.4
Czechia	46.7	4.5	2	8.7	19.0	5.7
Denmark	43.5	0.2	1.2	8.7	24.5	4.0
Estonia	47.9	5.5	2.3	9.0	20.0	6.3
Finland	45.3	3.9	1.3	10.5	20.0	6.2
France	50.4	4.8	1.1	6.8	26.5	4.9
Germany	46	4.7	1.4	9.4	30.0	5.8
Greece	64.4	7.0	0.7	10.5	24.0	7.4
Hungary	60.1	4.3	2.8	9.5	9.0	6.2
Ireland	67.4	5.0	0.6	7.0	12.5	5.5
Italy	66.8	4.0	1.1	7.2	24.0	4.8
Kosovo	59.0	10.3	2	18.2	10	12.4
Latvia	50.1	5.2	1.5	9.0	20	6.2
Lithuania	51.9	6.0	2.1	8.7	15.0	6.3
Luxembourg	46	4.7	1.7	7.0	24.9	5.0
Malta	67.7	4.7	1.4	7.2	35	4.8
Moldova	59.0	12	4.9	14.8	12	12.1
Montenegro	66.8	8.2	1.5	16.0	9	10.6
Netherlands	50.4	1.7	1.7	8.7	25.0	4.6
North Macedonia	66.4	7.6	1.6	15.0	10	9.9
Norway	40.1	3.4	2.2	9.2	22.0	5.4
Poland	45.2	6.1	2.1	8.7	19.0	6.5
Portugal	54.1	5.0	1.1	8.0	21.0	5.6
Romania	54.5	8.6	3.0	9.7	16.0	8.2
Russia	53.0	10.2	6.6	20.2	20.0	12.5
Serbia	61.5	8.3	4.1	19.7	15.0	11.7
Slovakia	47.2	5.2	1.7	10.4	21.0	6.7
Slovenia	52.9	5.0	1.2	9.0	19.0	6.1
Spain	54.4	4.9	1.3	6.9	25.0	5.1
Sweden	38.9	3.7	1.2	9.8	20.6	5.8
Switzerland	50.4	2.7	0.0	6.1	14.9	4.0
Turkey	96.52	19.0	10.9	17.0	25.0	15.2
Ukraine	59.70	17.7	11.7	18.6	18.0	15.9
United Kingdom	67.38	0.5	2.0	9.2	19.0	4.4

The analysis was limited to the reservoirs located within the coordinates 10°W, 45°E, 34°N, and 60°N. The suitable reservoirs were determined from the list available in the Global Reservoir and Dam Database (GRanD) v1.3 [60], applying the filters adopted by Spencer et al. [61]. GRanD is one of the most comprehensive databases on dams and was previously employed also by the World Bank to quantify the potential global FPV capacity [62]. The annual PV electricity yield generated by an FPV system (Y_F_) on each reservoir was calculated using the PVWatts DC power model [63] implemented in the pvlib python library [[64], [65]] and the procedure described in Ref. [19], bearing in mind that the most usual configuration of the FPV system is a fixed low-tilt (10° in this case) flat-plate monofacial module. The plane of array irradiance was determined from the hourly data available from CAMS radiation service [[66], [67], [68]] using the Perez model for diffuse irradiance [[69], [70], [71]], the model in Ref. [72] for ground-reflected irradiance and the incidence angle modifier described in Ref. [73]. Fixed 14 % losses and a 96 % efficient inverter were modeled, as outlined in Ref. [74].

**Fig. 1:**
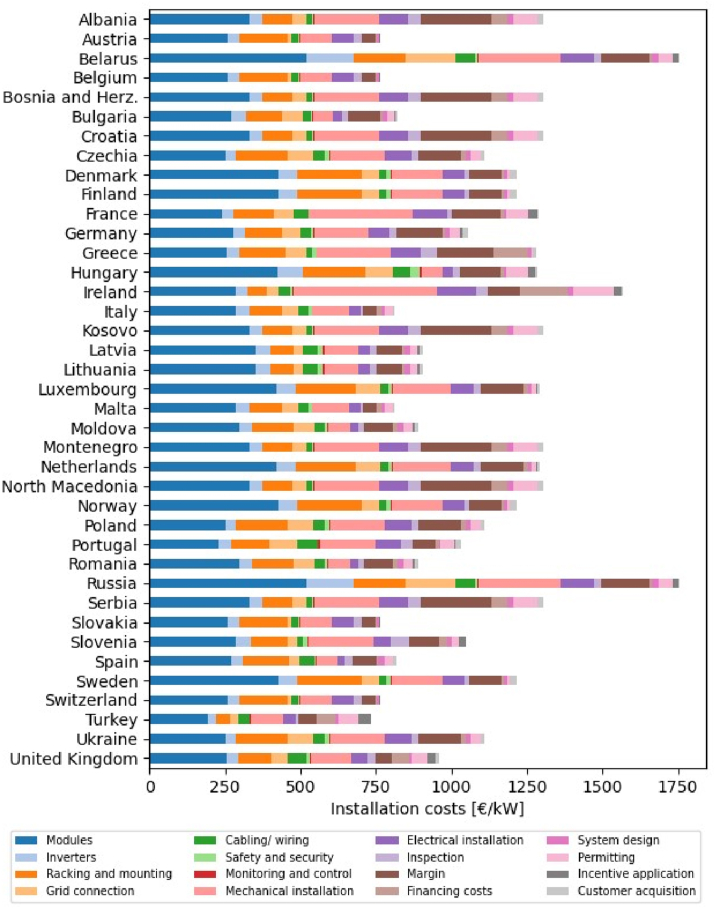
*CAPEX for FPV systems in 2022, estimated as in* Ref. [19] *from the data available in* Refs. [6,75].

All the upfront costs needed to install a PV system are grouped in the CAPEX (capital expenditure). These cover expenses ranging from the acquisition of the materials to the inspection costs and the developer profits. The various categories represent different percentages of the CAPEX, and their weights change with the country and with time. For this reason, one cannot just assume a fixed increase in CAPEX when moving from LPV to FPV, equal for all countries. Therefore, the FPV CAPEX and OMEX in this work have been calculated from the data available in the latest IRENA report on renewable power generation costs [6], from the information provided in Ref. [75] and through the methodology introduced in Ref. [19]. Specifically, IRENA reports typical country-specific CAPEX breakdowns for land-based photovoltaics [6], whereas the document published by NREL [75] shows how cost breakdowns change between similar LPV and FPV systems. This way, the information in the latter was employed to calculate cost category-specific “conversion factors”, to transform an LPV cost breakdown into an FPV cost breakdown. These conversion factors were then applied to the country-specific LPV cost breakdown in Ref. [6] to generate the country-specific FPV CAPEX breakdowns shown in Fig. 1. A similar procedure was employed to estimate each country's OMEX, starting from the LPV values reported in Ref. [6], and applying a similar conversion factor calculated from the data available in Ref. [75]. It should be noted that any value originally reported in US dollars ($) was converted into euros (€) using the average European Central Bank's conversion factor for 2022: 1.0530 $/€.

Usually, the CAPEX may be financed with debt (low-interest loans) or mixed financing, composed of a low-interest loan and own capital. In this work, mix financing has been considered. Specifically, 70 % of the amount is assumed to be borrowed at an annual loan interest (*i*_*l*_) and loan term (*N*_*l*_), as in Ref. [76]. The remaining amount is assumed to be own capital, so the annual retribution is given in the form of dividends (*d*_*s*_) and amortized at the end of the life cycle of the system. Consequently, the WACC varies depending on which financial resources are chosen to finance the CAPEX. The country-specific loan interest rates and nominal equity IRRs (*i*_*l*_ and *d*_*s*_) that have been employed in this work are reported in Table 1. The raw data employed for their calculation were sourced from Refs. [55,56] and from Ref. [58], respectively.

The average electricity prices were estimated for each country as a mean of 2010–2021 daily values and range from 40.1 €/MWh to 96.5 €/MWh (Table 1). As in Ref. [19], the historical daily prices were obtained from the databases of the Nominated Electricity Market Operators and of national and international economic service providers [[37], [38], [39], [40], [41], [42], [43], [44], [45], [46], [47], [48], [49], [50], [51], [52], [53], [54]]. The fact that electricity price varies with time has also been considered, even if it is always difficult to model because it is linked to the behavior of electricity markets. However, it seems that the rising trend of energy consumption—mainly due to emerging economies—will make the world's energy demand stronger. This fact, together with the increase in oil prices, is expected to push up electricity rates [77]. Therefore, an annual increase in electricity price has been assumed, set equal to the inflation rate of each considered country. The inflation was calculated, for each country, as the average of the 2010 to 2021 period, with values ranging from 0 % to 11.7 % (Table 1). The historical data were downloaded from those available on the World Bank database [57]. Similarly, also the annual increase rate of the operation and maintenance cost was assumed equal to the value of the annual inflation rates.

There are different income tax rates (T) depending on each country's regulations, sourced from Ref. [59] and also listed in Table 1. These rates have been applied to the cash inflow and outflow from the FPV system and to the asset depreciation. The depreciation for tax purposes is assumed linear and constant over a given period [10]. As in previous works [10,19], a maximum linear coefficient of 5 % with a depreciation period of 20 years was modeled for all countries.

As in Refs. [19,36], the following assumptions were adopted. Firstly, the discount rate (d) was set equal to the WACC. Secondly, the PV system lifetime (N) was set at 25 years. Thirdly, the system degradation was modeled as linear, with an annual rate of 0.75 %/year [78]. Lastly, any missing data was imputed with the corresponding value from a similar country.

### 3.3 Sensitivity analysis

A sensitivity analysis was carried out to determine how variations in the factors influencing the evaluation criteria (NPV, IRR, and LCOE) could affect the economic feasibility of FPV systems. In order to provide realistic results for the analysis, a reference scenario was defined for each country, based on the actual economic conditions listed in Table 1. Subsequently, variations have been made to the value of each parameter to evaluate their influence on the FPV economics. Following the approach employed in Refs. [14,79], variations from the base case scenarios ranging from −50 % to +50 %, with increments of 10 %, have been taken into account. The impact of the variation of an individual variable at a time is shown.

## 4 Results and discussion

The results of a comprehensive sensitivity analysis of the three economic criteria are presented in this section. First, the changes induced in the NPV, the IRR, and the LCOE as a function of the variations of the main techno-economic parameters are individually discussed in sub-sections 4.1, 4.2, 4.3. The results presented in these subsections are calculated as follows. The data shown in Fig. 1 and Table 1 are used to estimate the base case for each country. Subsequently, these parameters are systematically varied between −50 % and +50 % at 10 % increments to generate a thorough sensitivity analysis. Second, sub-section 4.4 presents a more in-depth discussion for five representative countries. In particular, equations that can be utilized to estimate the different criteria under various economic conditions are provided for these countries, whereas detailed results for the rest of the countries are reported in the Supplemental Material. Last, subsection 4.5 discusses the most effective mechanisms that could be put in place to financially support the deployment of FPV and summarizes the conclusions and limitations of the present work.

### 4.1 Net present value

In the reference conditions, the NPV is averagely positive in Turkey, Italy, and Spain. The NPV remains positive in Turkey and Italy also if the parameters are varied by no more than ±10 %. In Spain, on the other hand, the NPV can become negative even if some parameters are varied by ± 10 %. For instance, a 10 % increase in CAPEX or WACC or a 10 % decrease in average electricity price compared to reference conditions can lead to a negative NPV in the latter country.

Negative NPVs are observed, in reference conditions, for all the remaining countries. However, changes in a single parameter can lead to positive results in the NPV. This is the case, for example, of Portugal, Romania, and Switzerland if CAPEX is reduced by 30 % or if electricity prices grow by 30 %. In Bulgaria, an increase in price of 20 %, a reduction in CAPEX of 20 %, or a decrease in discount rate by 30 % would be sufficient to turn positive the NPV. In Ukraine, the price should increase by 40 % or the discount rate should decrease by 30 % to have NPV >0. Last, in the United Kingdom, the FPV would have a positive NPV if electricity prices were increased by 50 %. Only a reduction in CAPEX of 50 % would make the NPVs positive in Albania, Austria, Greece, Hungary, and Slovakia. It should be highlighted that, if changes occur simultaneously in multiple parameters, the required percentage changes could be lower than those needed for a single parameter.

**Fig. 2:**
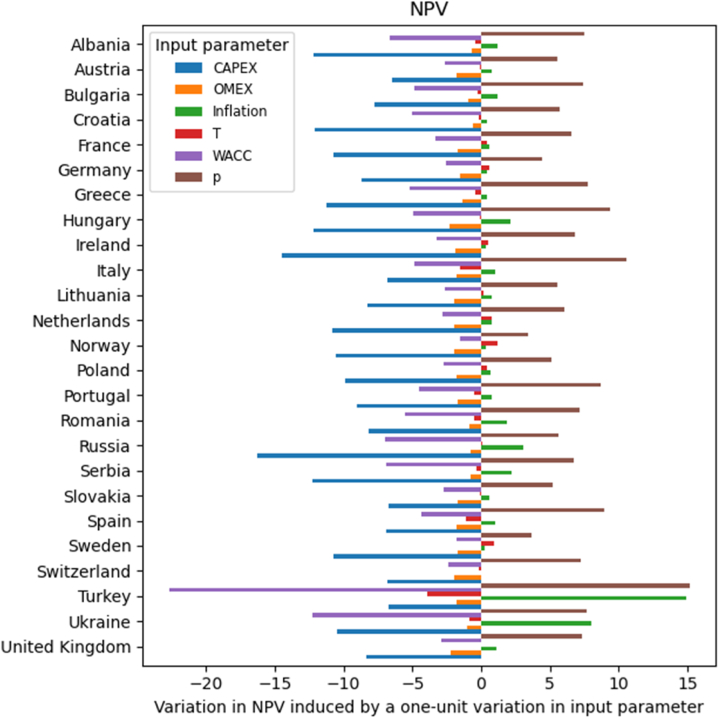
Variation in NPV [€/kW] induced by the various economic variables in the investigated countries.

Fig. 2 illustrates the impact of the investigated parameters' variations on NPV across different countries, assuming linear correlations. Increases in CAPEX, discount rate, and OMEX result in a reduction of the NPV, while increases in electricity prices and inflation rates have positive effects. The reason behind the results obtained for CAPEX, OMEX, and electricity prices is intuitive: higher costs reduce the revenues, whereas a higher remuneration for kWh increases the cash inflows. On the other hand, to understand the influence of the discount rate, one has to take into account that this rate represents the return used to discount future cash flows back to their present value. This rate often corresponds to the company's Weighted Average Cost of Capital (WACC), the required rate of return, or the hurdle rate that investors expect to earn relative to the risk of the investment. In this work, the discount rate is assumed to be equal to the WACC. Therefore, an increase in the cost of capital leads to higher financing costs, resulting in decreased cash flows. The inflation rate is, on the other hand, used to estimate the annual increase rates of electricity prices. For this reason, higher inflation results in an increased cash flow.

Last, it should be noted that taxes can have either positive or negative effects, depending on the conditions of each country. The negative effect occurs when the total tax payment exceeds the amount derived from the tax deduction through the depreciation mechanism. Conversely, the positive effect occurs when the tax payment is lower than the amount resulting from the tax deduction. In this last case, a proportion of these tax deductions will be applied in subsequent year(s) in which the tax payment exceeds the tax deduction. This positive effect is often observed where parameters such as yield, electricity price, or inflation have low values. For instance, a positive effect (i.e., a raise in NPV with a tax increase) is registered in France, Germany, Ireland, Lithuania, Netherlands, Norway, Poland, Russia, and Sweden, where the energy yields are lower compared to the Mediterranean countries. The calculation of after-tax cash flows (${\text{CF}}_{\text{after}-\text{tax}}$) can also further help to understand this outcome:

(7) $${\text{CF}}_{\text{after}-\text{tax}}={\text{CF}}_{\text{before}-\text{tax}}∙\left(1-T\right)+Dn∙T$$

The negative effect (i.e., decrease in NPV as a result of increased taxes) is achieved when the impact of the first term is greater than that induced by the second term (i.e., tax payment exceeds the amount derived from tax deduction), as in the case of Spain. The positive effect (i.e., increased NPV as a result of increased taxes) is on the other hand achieved when the impact of the first term is lower than that induced by tax depreciation, as in the case of France.

Overall, Fig. 2 indicates that tax rate, inflation rate, and OMEX have a relatively limited impact on the NPV, whereas CAPEX and electricity price have a dominant influence, leading to substantial variations. The impact of the discount rate is also among the lowest in most countries. However, it is more significant in Turkey and Ukraine. In the former country, in particular, its impact surpasses that of CAPEX. This is due to the highest WACC values registered in these two countries (15.2 % in Turkey and 15.9 % in Ukraine). Indeed, high WACC values result in high discount rates, causing a negative impact on the NPV. Similarly, also other countries such as Albania, Romania, Russia, and Serbia, whose WACC values range from 8.2 % to 12.5 %, experience a markedly negative impact of the discount rate on the NPV, even if to a lesser extent than Turkey and Ukraine.

**Fig. 3:**
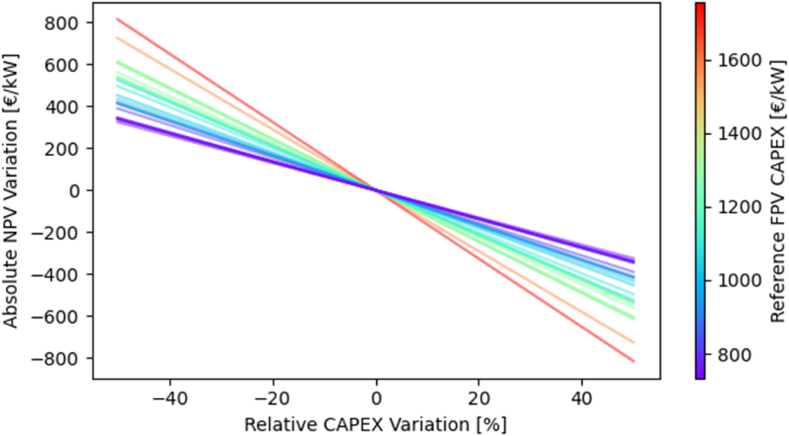
Correlations between relative CAPEX variation and induced absolute variation in NPV. Each line represents a country and is colored according to the estimated reference FPV CAPEX.

As mentioned, the CAPEX is, in most cases, the dominant factor affecting the NPV (Fig. 2). This is particularly important because, since 2010, the CAPEX of traditional LPV has dropped by 90 % [6]. While most of this reduction was due to a decrease in the cost of PV modules, also the economy of scale in the balance of system (BoS) contributed to it. Similar scale effects can be expected, in the future, for the BoS of FPV, whose supply chain is not fully developed yet. In light of this expected drop in installation costs, the correlation between CAPEX and NPV was more in-depth investigated. In particular, a linear correlation was found between the relative variation in CAPEX and the consequent absolute variation in NPV, as shown in Fig. 3. The slope of the correlation varies in each country, according to the magnitude of the reference CAPEX. The average value is −9.8 €/kW/% with extremes of −6.5 €/kW/% in Austria and −16.3 €/kW/% in Russia. In first approximation, the slope can be considered proportional to the reference CAPEX (i.e., the CAPEX in the base case scenario), and estimated using the following equation (R^2^ = 97.2 %):

(8) $$\Delta NPV=-0.009\;∙\;CAPEX_{ref}+0.48$$

### 4.2 Internal rate of return

In the reference conditions, the IRR is positive, on average, in most of the countries. Variations by ± 50 % of the economic conditions would not affect the results in Turkey, and Ukraine. Conversely, a more sensitive response is observed in a number of countries, namely Albania, Austria, Bulgaria, Croatia, Greece, Hungary, Italy, Portugal, Romania, Russia, Serbia, Slovakia, Spain, and Switzerland, where the IRR remains positive only within a narrower parameter fluctuation of ±10 %. In Poland, the IRR would turn positive if CAPEX is reduced by 30 %, or price increases by 20 %. In the Netherlands and Germany, decreases in CAPEX of 30 % or increases in price by 30 % are needed. In Ireland, increases in price >40 % would turn the IRR positive. Remarkably, in Sweden and Norway, the IRR remains non-positive even when factors are varied by up to 50 %.

**Fig. 4:**
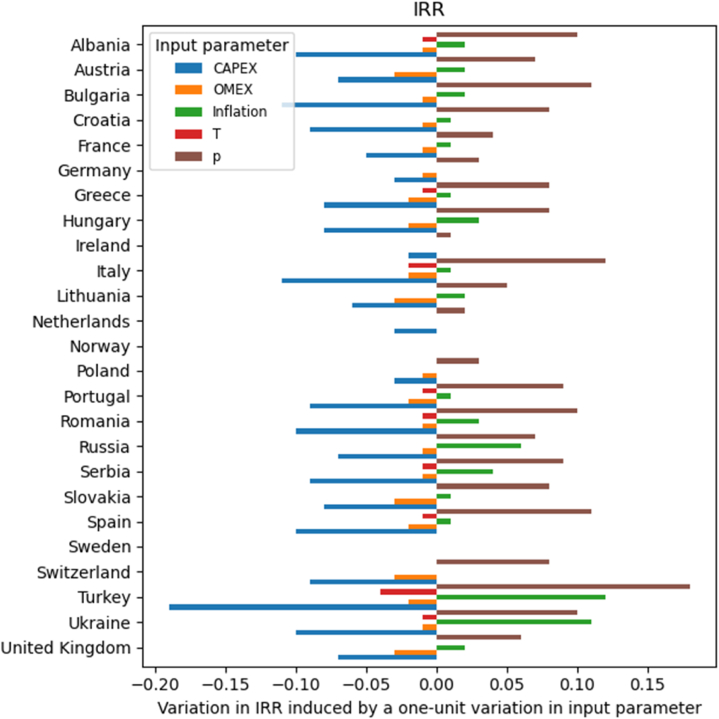
Variation in IRR [%] induced by the various economic variables in the investigated countries.

Fig. 4 shows the variation that the investigated parameters induce in the IRR across the various countries. As shown in eq. (5), the IRR criterion has been defined as the discount rate (*d*) that makes the NPV equation equal to zero. Therefore, the discount rate cannot be evaluated in this subsection.

CAPEX, OMEX, and tax rates have a negative effect on the IRR, while increases in electricity prices and inflation rates have a positive effect. The results for CAPEX, OMEX, and electricity prices are intuitive: higher costs lead to lower profits, whereas higher remuneration for kWh increases the cash inflows. The electricity price has the highest impact, followed by CAPEX and, in some countries, by inflation.

A positive IRR is a necessary but not sufficient condition for a sustainable investment. Indeed, to return a profit, the IRR should be positive and greater than the WACC. Even if the IRR is positive in most countries, it is greater than the WACC only in Italy, Spain, and Turkey. In the first and last countries, the criterion is true only if the input parameters are varied by no more than ±10 %. On the other hand, in Spain, the IRR becomes lower than the WACC if the CAPEX increases by 10 % or the price decreases by 10 %. Additionally, under some conditions, IRR can be greater than WACC in Albania, Austria, Bulgaria, Greece, Hungary, Portugal, Romania, Slovakia, Switzerland, Ukraine, and the United Kingdom.

### 4.3 Levelized cost of electricity

**Fig. 5:**
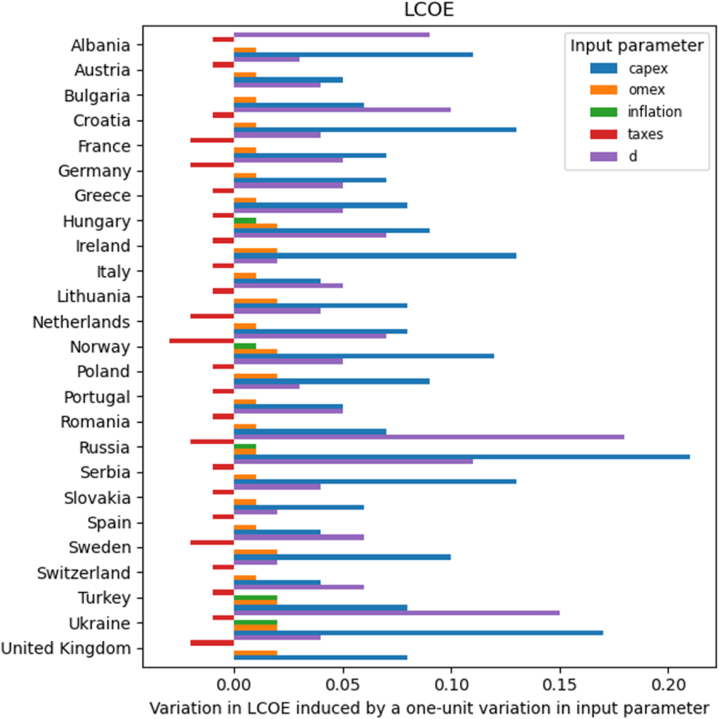
Variation in LCOE [€_cent_/kWh] induced by the various economic variables in the investigated countries.

Fig. 5 shows the variation that the investigated parameters induce in the LCOE across the various countries. An increase in all parameters but taxes raises the LCOE, therefore reducing the cost-competitiveness of this technology. The lowering effect of the taxes on the LCOE (i.e., a decrease in LCOE induced by an increase in taxes) is due to the PV tax depreciation. This is a mechanism that helps owners keep their installation costs down. FPV owners/investors can use depreciation to recover their asset's costs as the equipment's value declines throughout the system's lifetime. Therefore, tax depreciation saves on tax payments and contributes to reducing the cost of the FPV over its useful life.

Even in this case, the results for CAPEX and OMEX are intuitive: higher initial or annual expenses for the same energy yield increase the overall cost of electricity. In order to understand the influence of the other two variables, one has to remember that the value of the inflation rate is assumed to be equal to the annual increase in OMEX and that the discount rate is assumed equal to the WACC. Therefore, increases in inflation or discount rates result in higher costs. Overall, it is found that the magnitude of the variation induced by tax rate, inflation rate, and OMEX on the LCOE is limited, whereas CAPEX and discount rate are dominant. In particular, the impact of CAPEX is always bigger than that of the discount rate.

The LCOE can be an effective tool to evaluate how likely is a technology to be included in the energy mix of a country. This can, indeed, be estimated by comparing the LCOE and the electricity price: the LCOE of a source should be lower than the electricity price in the energy market. For FPV, this condition is respected only in Italy and Spain. In the former country, the criterion is true even if any of the economic conditions vary by ± 50 %. In Spain, this is true only if the parameters are varied by no more than ±10 %. On the other hand, the LCOE can become lower than the electricity price if, in Switzerland, the CAPEX decreases by 10 %, or, in Turkey, if CAPEX, OMEX, WACC, or inflation lower by 10 %.

Additionally, under some conditions, the LCOE can become lower than the electricity price in Bulgaria, Greece, and Portugal. The CAPEX has to be reduced by at least 30 % in the first two countries. In Portugal, the criterion is met if the CAPEX or discount rate is reduced by 20 % at least. More severe reductions in CAPEX are needed in the United Kingdom, Austria, Romania (40 %) France, Slovakia, and Albania (50 %).

**Fig. 6:**
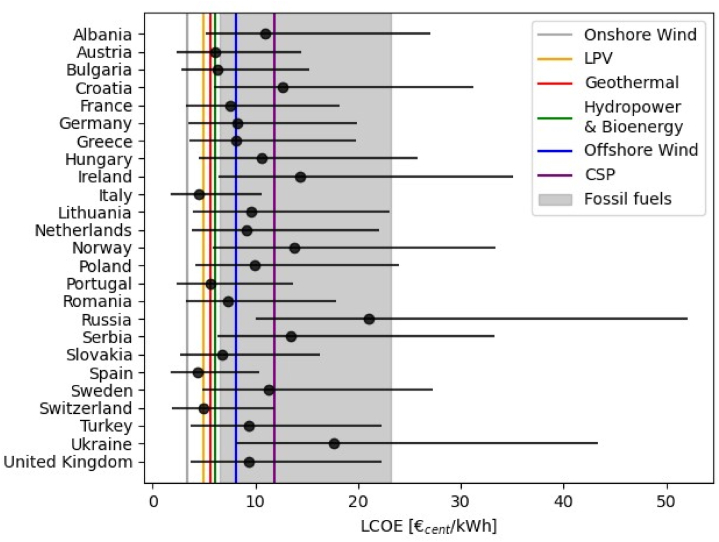
*Comparison between country-specific LCOEs estimated for FPV in the baseline scenario and the global LCOEs reported for various renewable and non-renewable sources in* 2022 by Ref. [6]. *Hydropower and bioenergy have the same average LCOE. The round markers show the average LCOE for FPV calculated in each country in the baseline scenarios. The error bars show the LCOE values for FPV when CAPEX is varied from -50 % to +50 %.*

The LCOE can also be used to compare the cost-competitiveness of FPV with that of different technologies. Fig. 6 shows the country-specific LCOEs obtained for FPV in the baseline scenario of this analysis (black markers) and the global average values reported by IRENA [6] for 2022 for seven renewable sources (vertical colored lines), along with a fossil fuels’ cost range (grey shaded area). The bars around the black markers show the variation in LCOE experienced when the CAPEX is varied by −50 % to +50 %.

As can be seen, the cost-competitiveness is significantly location-dependent, with the lowest values achieved in high-irradiance and relatively low CAPEX countries, such as Italy and Spain. As discussed, in Ref. [30], in these southern countries, FPV is benefitted by an averagely higher position of the Sun (which limits the low-tilt induced losses) and by higher ambient temperatures, whose negative effect is reduced thanks to the improved FPV cooling. In several other countries, such as Germany and Greece, the combination of low-to-intermediate CAPEX and good irradiance conditions leads to LCOEs similar to or below that of offshore wind. In the remaining countries, the high CAPEX and the limited irradiance conditions result in higher costs of energy, with extreme values registered in Russia. Fig. 6 also depicts the significant impact that variations in CAPEX can have on the LCOE of FPV. Specifically, reductions in CAPEX, achievable through the development of the supply chain [80] or through specific support mechanisms, can increase the cost-effectiveness of FPV, making it as cheap as LPV in several countries, and even cost-competitive with wind energy in some areas.

### 4.4 Case studies

**Fig. 7:**
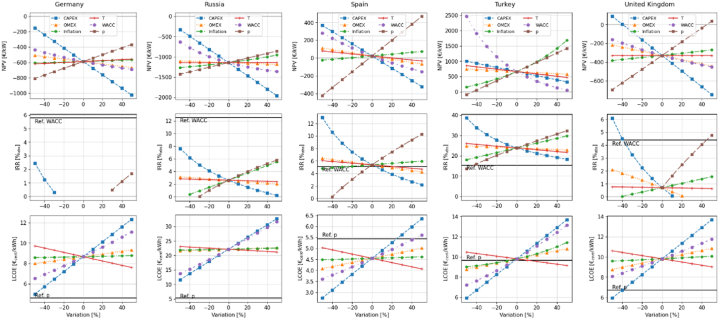
Absolute variations in NPV, IRR, and LCOE values induced by the relative variations of the different inputs. Only conditions for which IRR >0 % are shown. The IRR plots also show the reference WACC value for each country. Similarly, the LCOE plots show the reference electricity price (“Ref. p”) for each country.

This work presents an economic analysis of twenty-five European countries, whose most relevant results have been presented in Sections 4.1, 4.2, 4.3. In the present section, a regression curve is determined for each economic criterion and independent variable in the sensitivity analysis. This involves finding the mathematical function that best fits the correlation between each criterion and each variable. For conciseness, the results obtained for a limited group of five countries are shown in Fig. 7 and Table 2. However, analogous plots for all the other countries can be found in the Supplemental Material.

**Table 2:** Best fit equations.

		NPV	IRR	LCOE
Germany	CAPEX	y = −8.6943*x-586.8431 (R^2^: 99 %)	y = −0.1068*x-2.9346 (R^2^: 99 %)	y = 0.0733*x+8.6556 (R^2^: 100 %)
	OMEX	y = −1.5707*x-586.8431 (R^2^: 100 %)		y = 0.0132*x+8.6556 (R^2^: 99 %)
	inflation	y = 0.4008*x-586.459 (R^2^: 99 %)		y = 0.002*x+8.6576 (R^2^: 99 %)
	taxes	y = 0.6316*x-586.8431 (R^2^: 100 %)		y = −0.0212*x+8.6556 (R^2^: 99 %)
	d	y = −2.5264*x-576.5597 (R^2^: 98 %)		y = 0.0459*x+8.714 (R^2^: 99 %)
	p	y = 4.3966*x-586.8431 (R^2^: 100 %)	y = 0.0598*x-1.2979 (R^2^: 99 %)	
Russia	CAPEX	y = −16.2829*x-1142.7508 (R^2^: 100 %)	y = 0.0005*x^2–0.0705*x+2.5853 (R^2^: 99 %)	y = 0.211*x+22.1375 (R^2^: 100 %)
	OMEX	y = −0.8046*x-1142.7508 (R^2^: 99 %)	y = −0.0109*x+2.6082 (R^2^: 99 %)	y = 0.0104*x+22.1375 (R^2^: 100 %)
	inflation	y = 3.0384*x-1130.1281 (R^2^: 98 %)	y = 0.0568*x+2.6337 (R^2^: 99 %)	y = 0.0068*x+22.1665 (R^2^: 98 %)
	taxes	y = 0.0573*x-1142.7508 (R^2^: 100 %)	y = −0.0043*x+2.6115 (R^2^: 99 %)	y = −0.0191*x+22.1375 (R^2^: 100 %)
	d	y = 0.058*x^2–6.9871*x-1144.43 (R^2^: 99 %)		y = 0.1796*x+22.3481 (R^2^: 99 %)
	p	y = 5.66*x-1142.7508 (R^2^: 100 %)	y = 0.0707*x+2.4561 (R^2^: 99 %)	
Spain	CAPEX	y = −6.9203*x+23.2287 (R^2^: 99 %)	y = 0.0009*x^2–0.1009*x+5.3256 (R^2^: 99 %)	y = 0.0361*x+4.547 (R^2^: 100 %)
	OMEX	y = −1.7906*x+23.2287 (R^2^: 99 %)	y = −0.0219*x+5.3713 (R^2^: 99 %)	y = 0.0093*x+4.547 (R^2^: 100 %)
	inflation	y = 0.9783*x+24.1208 (R^2^: 99 %)	y = 0.0118*x+5.3866 (R^2^: 99 %)	y = 0.0013*x+4.5482 (R^2^: 99 %)
	taxes	y = −1.12*x+23.2287 (R^2^: 100 %)	y = −0.0136*x+5.3773 (R^2^: 99 %)	y = −0.0097*x+4.547 (R^2^: 99 %)
	d	y = −4.3316*x+39.7883 (R^2^: 98 %)		y = 0.0201*x+4.5714 (R^2^: 99 %)
	p	y = 8.9432*x+23.2287 (R^2^: 100 %)	y = 0.1097*x+5.1252 (R^2^: 99 %)	
Turkey	CAPEX	y = −6.7674*x+660.7269 (R^2^: 100 %)	y = 0.0018*x^2–0.1887*x+23.7235 (R^2^: 99 %)	y = 0.0777*x+9.7985 (R^2^: 100 %)
	OMEX	y = −1.7666*x+660.7269 (R^2^: 99 %)	y = −0.0203*x+23.8597 (R^2^: 99 %)	y = 0.0203*x+9.7985 (R^2^: 100 %)
	inflation	y = 0.103*x^2 + 14.9158*x+659.1578 (R^2^: 99 %)	y = 0.1175*x+23.8789 (R^2^: 99 %)	y = 0.0002*x^2 + 0.0236*x+9.7959 (R^2^: 99 %)
	taxes	y = −3.8906*x+660.7269 (R^2^: 100 %)	y = −0.0442*x+23.8395 (R^2^: 99 %)	y = −0.0133*x+9.7985 (R^2^: 100 %)
	d	y = 0.2392*x^2–22.6967*x+650.3107 (R^2^: 99 %)		y = 0.0599*x+9.9429 (R^2^: 99 %)
	p	y = 15.1413*x+660.7269 (R^2^: 100 %)	y = 0.1841*x+23.5405 (R^2^: 99 %)	
United Kingdom	CAPEX	y = −8.3814*x-329.4537 (R^2^: 100 %)	y = 0.0009*x^2–0.0602*x+0.6562 (R^2^: 99 %)	y = 0.0773*x+9.8149 (R^2^: 100 %)
	OMEX	y = −2.259*x-329.4537 (R^2^: 100 %)	y = −0.0287*x+0.6935 (R^2^: 99 %)	y = 0.0208*x+9.8149 (R^2^: 100 %)
	inflation	y = 1.1247*x-327.8345 (R^2^: 99 %)	y = 0.0169*x+0.7086 (R^2^: 99 %)	y = 0.0048*x+9.822 (R^2^: 99 %)
	taxes	y = 0.0003*x-329.4537 (R^2^: 100 %)	y = −0.0013*x+0.7056 (R^2^: 99 %)	y = −0.0159*x+9.8149 (R^2^: 99 %)
	d	y = −2.9163*x-319.5453 (R^2^: 99 %)		y = 0.037*x+9.857 (R^2^: 99 %)
	p	y = 7.3459*x-329.4537 (R^2^: 100 %)	y = 0.0811*x+0.7911 (R^2^: 99 %)	

The selected five countries are Germany, Russia, Spain, Turkey, and the United Kingdom, and have been chosen to represent a variety of conditions. Germany is one of the countries with the largest solar capacity in Europe [6]. Russia is the country with the largest water surface in the investigated region [30] and has also the highest CAPEX among the investigated countries (Fig. 1). Spain and Turkey are two of the three countries with a positive NPV in reference conditions, as detailed in Section 4.1. Spain and Turkey have, indeed, some of the highest energy yields in Europe [19], which positively impact the economic returns of FPV. Additionally, Spain has low CAPEX and WACC (Table 1), which minimize the costs associated with the installation of an FPV system, whereas Turkey has high electricity prices and inflation (Table 1), which maximize the economic remuneration per unit of energy generated. Last, the United Kingdom is the first European country per installed FPV capacity [81] and is characterized by lower energy yields and WACC compared to other investigated countries (Table 1).

The degree of sensitivity of a criterion to the input variable is reflected in the steepness of the lines in Fig. 7. These lines have been fitted using a linear or a second-order regression, with the latter employed only when the R^2^ from linear regression fell below 98 %. The resulting equations, along with their respective R^2^ values, are detailed in Table 2. These can be immediately applied to estimate how the variation of the parameters involved in the computation of NPV, IRR, and LCOE criteria affects the outcome.

The plots in Fig. 7 reveal some interesting insights. Notably, Turkey is the only country where the WACC can exert a more substantial, whether positive or negative, influence on the NPV than the CAPEX. The reason for this result was explained earlier and relies on the high WACC value that the country has in the reference conditions (Table 1).

In Germany and Russia, the NPV remains negative even when factors are varied by up to ±50 %. In addition, the IRR values are lower than WACC even when parameters are varied by up to ±50 %. Therefore, FPV systems in these two countries are not feasible from an economic standpoint under the present conditions. Additionally, the LCOE values are higher than the average electricity price, indicating that FPV systems are not cost-competitive when compared to the average price of electricity. Even in this case, the sensitivity analysis does not return any cost-effective scenario for FPV (i.e., LCOE < p) in these countries.

In Spain and Turkey, the NPV remains positive and the IRR is higher than WACC in the base case and under certain economic conditions. It is noteworthy that, in Turkey, the NPV is positive and IRR values are higher than the WACC even when factors vary by up to 50 %, except for variations of −50 % of the average electricity price. Therefore, FPV systems in this country are feasible from an economic standpoint in the base case scenario and also under certain economic conditions. On the other hand, in Spain, LCOE values are lower than the average electricity price, making FPV cost competitive even when factors vary by up to 50 %, except for variations of 50 % in the discount rate and of more than 20 % in CAPEX. On the other hand, as can be seen, while the IRR is consistently greater than the WACC, in Turkey the LCOE is slightly lower than the electricity price in the baseline condition. Therefore, even if potentially profitable (thanks to the high electricity prices), FPV is not yet fully cost-competitive, because of the high cost of electricity. Reduction in CAPEX and WACC could however significantly reduce the LCOE, increasing the cost-competitiveness of FPV in the country.

In the United Kingdom, the NPV remains negative and the IRR is lower than the WACC, except for variations greater than −40 % in CAPEX and higher than 40 % of the average electricity price. Moreover, FPV systems are not cost-competitive, as the LCOE values are higher than the average electricity price, except for CAPEX variations greater than −40 %. Therefore, favorable conditions for FPV (i.e., LCOE < p and IRR > WACC) could be achieved in the United Kingdom if the CAPEX is substantially reduced by 40 % or more.

In Germany and the United Kingdom, the parameters can be ranked as follows, from highest to lowest impact on NPV and IRR: CAPEX, average electricity price, discount rate, OMEX, inflation rate, and tax. With respect to LCOE, in Germany, the hierarchy is CAPEX, rate discounted, tax rate, OMEX, and rate inflation. In the United Kingdom, the sequence is CAPEX, discount rate, OMEX, tax rate, and inflation rate.

In Russia, the influential factors on NPV and IRR follow this descending order: CAPEX, discount rate, average electricity price, inflation rate, OMEX, and tax. For LCOE, the hierarchy is as follows: CAPEX, discount rate, tax rate, OMEX, and inflation rate, with inflation rate and OMEX exerting a similar impact.

In Spain, the influential factors, ranked in descending order, on NPV and IRR are average electricity price, CAPEX, discount rate, OMEX, inflation rate, and tax rate, with inflation rate and tax having a similar impact. For LCOE, the sequence is as follows: CAPEX, discount rate, tax rate, OMEX, and inflation rate, with inflation rate and tax exerting a similar effect.

For Turkey, the parameters are ordered, concerning their impact on the NPV, as follows: discount rate, average electricity price, inflation rate, CAPEX, tax, and OMEX. For the IRR, the sequence is CAPEX, electricity price, inflation rate, tax rate, and OMEX. Last, concerning the LCOE the influential factors follow this order: CAPEX, discount rate, inflation rate, OMEX, and tax rate.

### 4.5 Discussion

The results obtained for the 25 investigated countries reveal that CAPEX and WACC have the greatest impact on LCOE, whereas OMEX, inflation, and income tax rate have a comparatively lower impact. Consequently, in countries where FPV is not yet cost-competitive (i.e., LCOE > p), strategic interventions targeting CAPEX and WACC are recommended. Support mechanisms could consist of grants or direct capital subsidies, aimed at decreasing the CAPEX, for example. Alternatively, low-interest and long-term loans could be put in place to decrease the WACC. Additionally, mechanisms such as income tax credits (i.e., reductions in taxes that taxpayers owe) can be used, even if these alone are expected to have a lower impact on the FPV economics compared to the previous ones.

Additionally, CAPEX, electricity price, and energy yield exert the greatest effect on IRR, whereas OMEX, inflation, and income tax rate have the least influence. This means that, in countries where FPV is not profitable (i.e., IRR < WACC), support mechanisms such as Feed-in Premiums (FiP), where FPV energy producers receive a remuneration above the market electricity price, could be established. Similar support mechanisms can be applied to increase the NPV of FPV, as this is predominantly influenced by CAPEX, electricity price, energy yield, and WACC.

An example of effective support mechanisms can be provided by analyzing the results of Spain (Fig. 7), where FPV is cost-competitive since the LCOE (4.6 €_cents_/kWh) is lower than the price of electricity (5.4 €_cents_/kWh). However, the IRR (5.4 %) is approximately equal to the WACC (5.1 %) and the NPV is relatively small (23 €/kW). Consequently, the investment is at the limit of economic viability and therefore unlikely attractive to investors. However, if subsidies are introduced to reduce the CAPEX by 20 %, FPV would reach an IRR of 7.5 %, a value greater than the WACC, and the NPV would reach a positive and sufficient value (161 €/kWh). In these conditions, FPV would become economically feasible, and the LCOE would lower to 3.8 €_cents_/kWh. A similar feasibility condition could be achieved through different support mechanisms, such as Feed-in Premiums for example. Specifically, if the electricity price paid to the FPV owners in Spain was increased by 20 %, FPV would reach an IRR of 7.5 % (IRR > WACC) and NPV a value of 202 €/kWh.

Overall, this work highlights the influence that variable economic conditions can have on the cost and profitability of FPV. As shown, the results can be used to identify initiatives and scenarios that can favor or hinder the deployment of this innovative renewable energy technology. However, it must be acknowledged that there is at least an additional parameter that influences the economics of FPV: the energy yield. Nonetheless, even if not explicitly mentioned, the effect of a variation in energy yield on NPV and IRR is the same as that of the electricity price, as the product of these two variables is employed to calculate these profitability criteria.

The present work considers the yield of a fixed 10° tilted monofacial FPV system, currently the most common configuration [20]. However, several alternative geometries and designs have already been proposed, and these can affect the energy yield. Higher tilt angles, for example, can lead to higher energy conversions, but at the same time can be expected to increase the installation costs [82]. Similar effects can be anticipated for improved cooling and more efficient or bifacial modules, but their costs and benefits cannot be yet fully quantified. For this reason, further research is needed to comprehend the influence of innovations and alternative designs, such as higher tilt angles or bifacial modules, on both FPV costs and profitability.

Given these considerations, the present work solely focused on a specific FPV configuration, analyzing variations induced by economic conditions only. This approach allowed quantifying the effects of the variable economic scenario in which FPV is being deployed and made it possible to identify equations for modeling these impacts. The results and equations derived from this study can immediately serve as practical tools for investors, operators, and decision-makers to predict the impacts of diverse economic scenarios on the FPV deployment. The analysis, indeed, applied the same systematic approach to suitable reservoirs in 25 European countries, considering their specific weather and economic conditions. The results obtained for 5 of these countries were discussed in detail in section 4.4, whereas the most important results for the remaining country were mentioned in 1, 2, 3, 4 and all the plots obtained from the sensitivity analysis are included in the Supplemental Material. Additionally, the models employed in this work are described, as well as the data sources, so that the analysis can be reproduced for additional locations and different FPV configurations and designs.

## 5 Conclusions

This paper evaluates the effects that changes in economic conditions can have on the cost-competitiveness and profitability of FPV across 25 European countries. The study starts with the analysis of the actual conditions in each country and then analyses the variations in net present value, internal rate of return, and levelized cost of electricity induced by variable economic conditions.

The results indicate that parameters such as capital expenditure (CAPEX), average electricity price, and discount rate have a significant impact on NPV, IRR, and LCOE. For this reason, support mechanisms such as grants, direct capital subsidies, low-interest loans, and/or feed-in tariffs are recommended to lower the costs and/or increase the profitability of FPV. Indeed, a 1 % variation in CAPEX could lead to an average increase in NPV of almost 10€/kW. This is particularly significant, as substantial decreases in installation costs can be expected in the future, as the FPV market grows and the economy of scale kicks in. Other factors, such as OMEX, inflation rate, and tax rate exert a relatively lower effect on NPV, IRR, and LCOE. Interestingly, an increase in taxes can produce either a positive or a negative effect on the economic viability of FPV, depending on the magnitude of parameters such as the energy yield and the CAPEX.

The present work was limited to European countries and analyzed the economics of a south-facing 10° tilt monofacial FPV configuration. However, the proposed methodology could be applied in reproducibility studies to additional locations and/or conditions. Further research is indeed needed to understand how alternative designs, such as higher tilt angles or bifacial modules, could impact both the costs and the profitability of FPV.

## Data availability statement

Data will be made available on request.

## CRediT authorship contribution statement

**Leonardo Micheli:** Writing – original draft, Visualization, Software, Methodology, Formal analysis, Data curation. **Fredy A. Sepúlveda-Vélez:** Writing – review & editing. **Diego L. Talavera:** Writing – original draft, Validation, Methodology, Investigation, Data curation, Conceptualization.

## Declaration of generative AI and AI-assisted technologies in the writing process

During the preparation of this work the authors used ChatGPT by OpenAI in order to improve spelling, grammar, clarity, and general editing. After using this tool, the authors reviewed and edited the content as needed and take full responsibility for the content of the publication.

## Declaration of competing interest

The authors declare that they have no known competing financial interests or personal relationships that could have appeared to influence the work reported in this paper.
