# Supplementary figures and images for: Impact of variable economic conditions on the cost of energy and the economic viability of floating photovoltaics
Source: Heliyon. 2024 Jun 4;10(12):e32354. doi: 10.1016/j.heliyon.2024.e32354 (PMC11237847; doi:10.1016/j.heliyon.2024.e32354)

# Supplemental Material

**
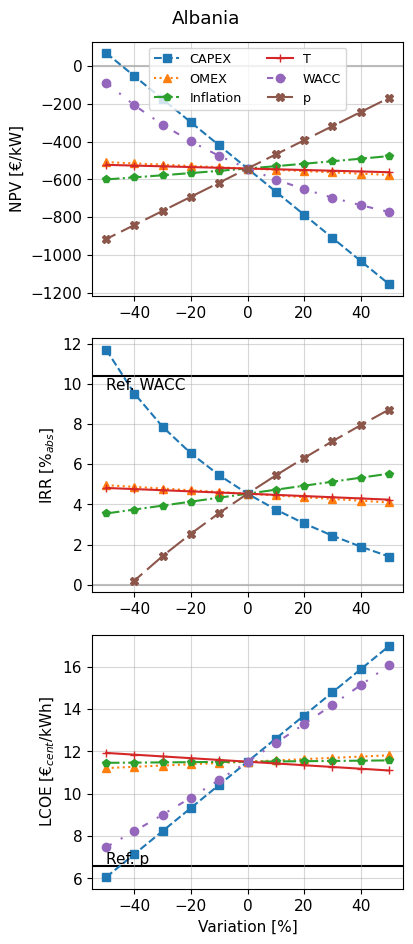

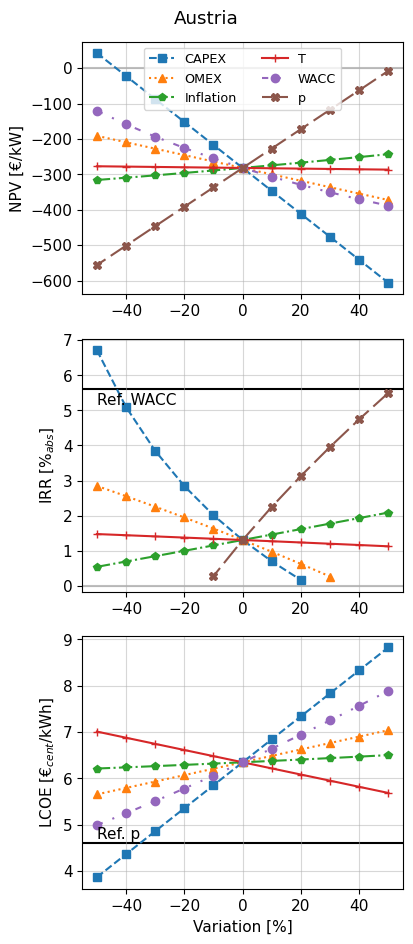

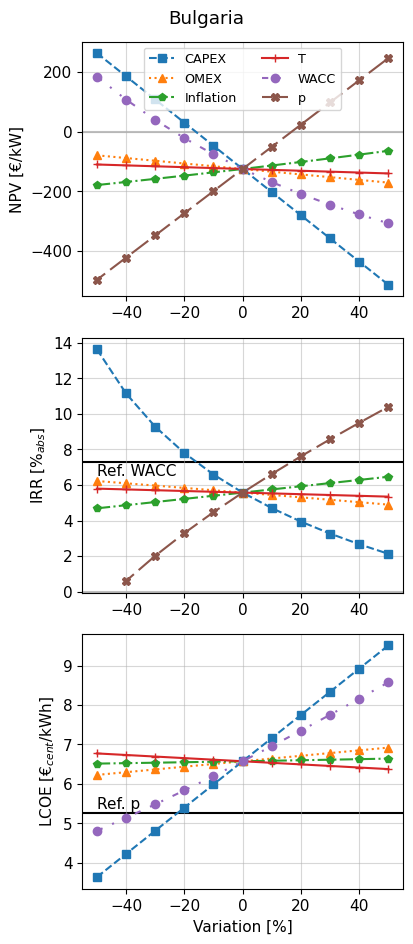

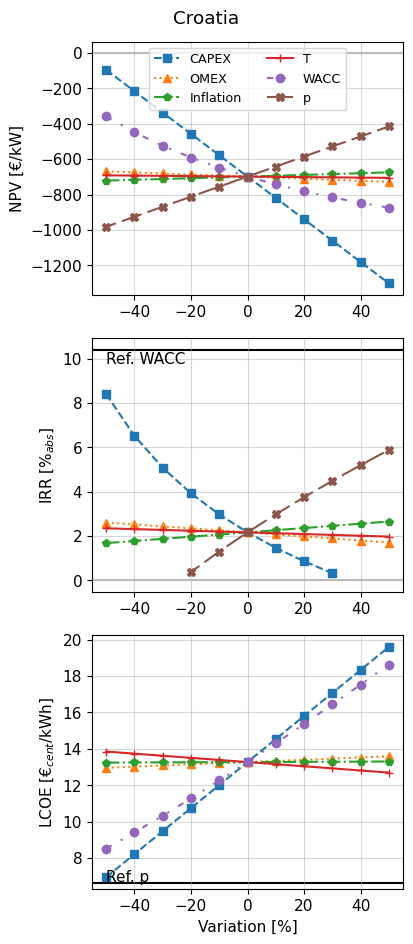

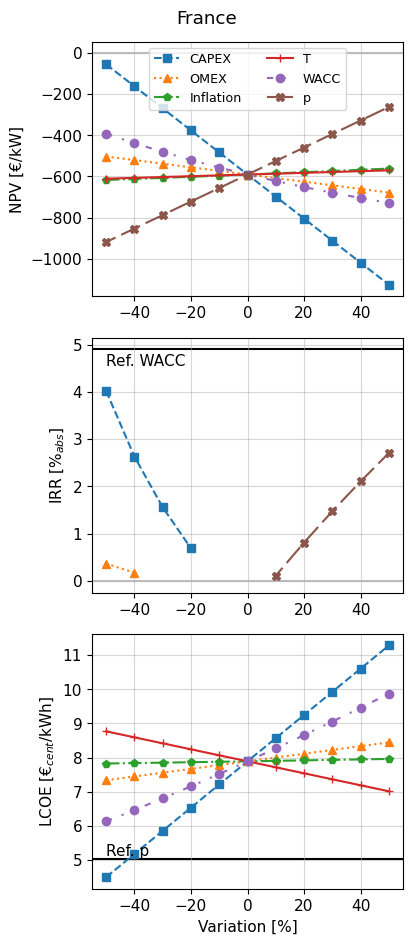

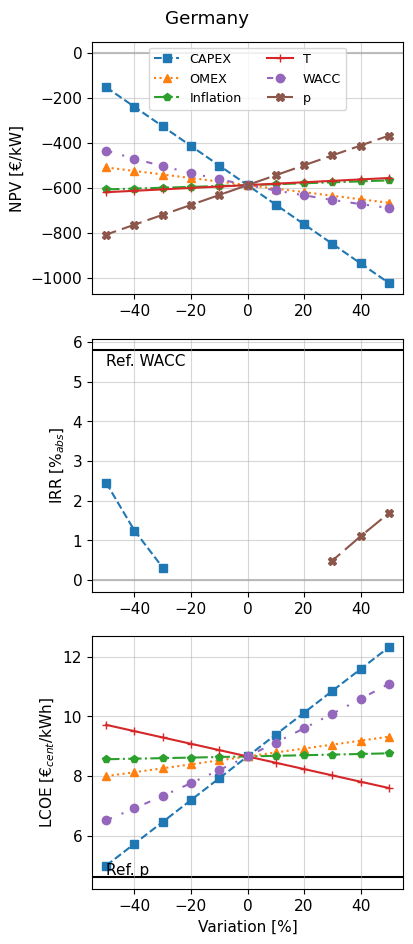

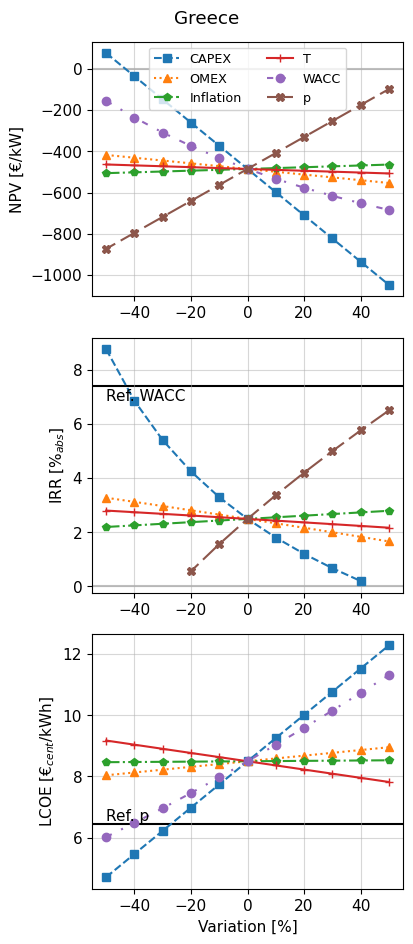

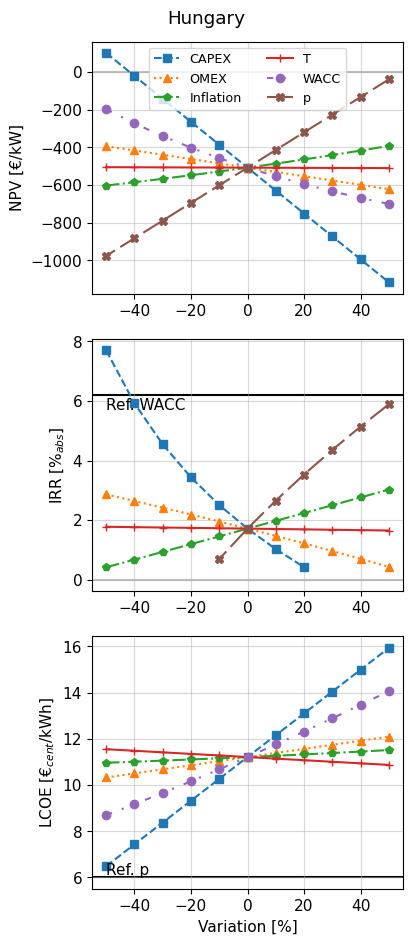
**

**
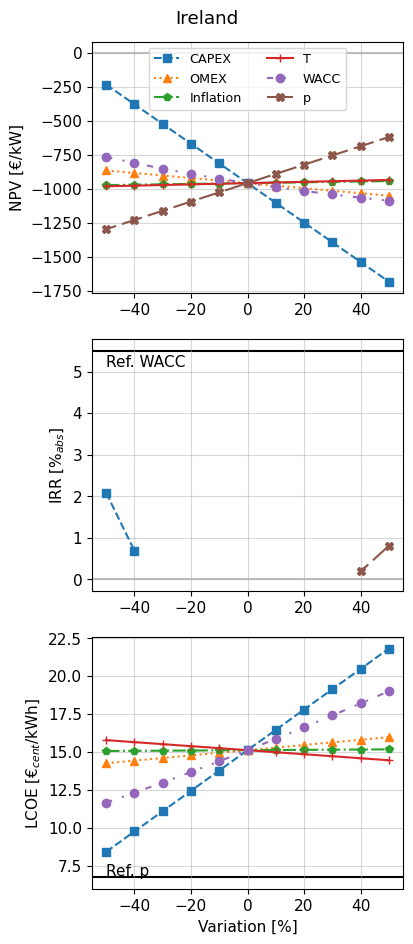

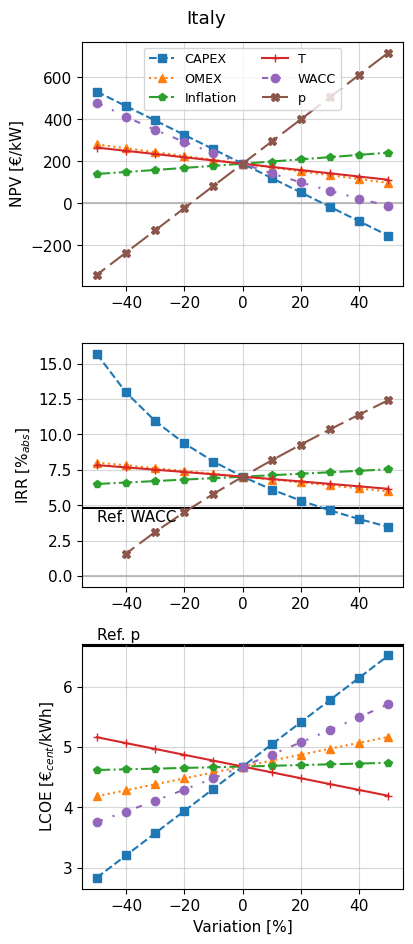

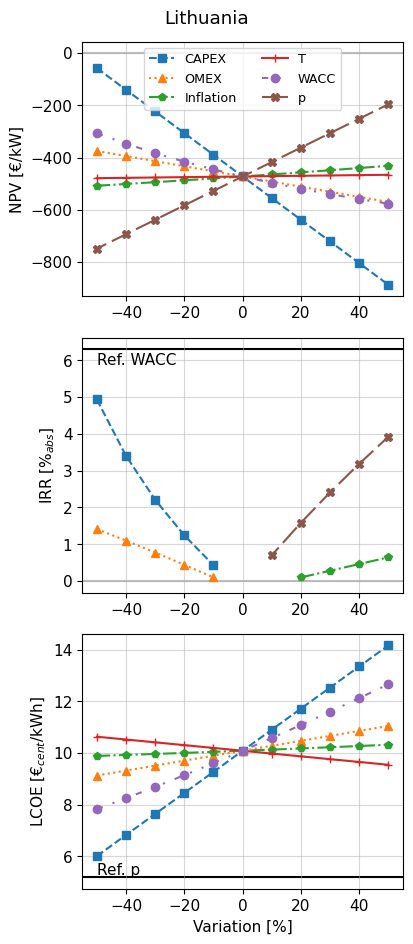

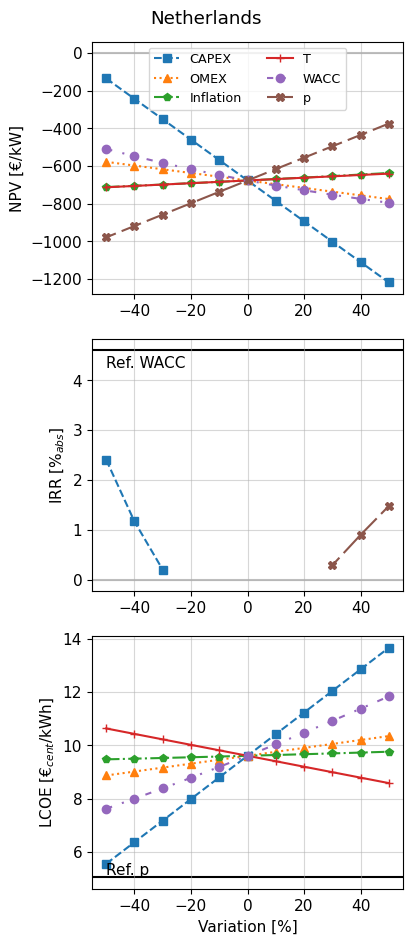

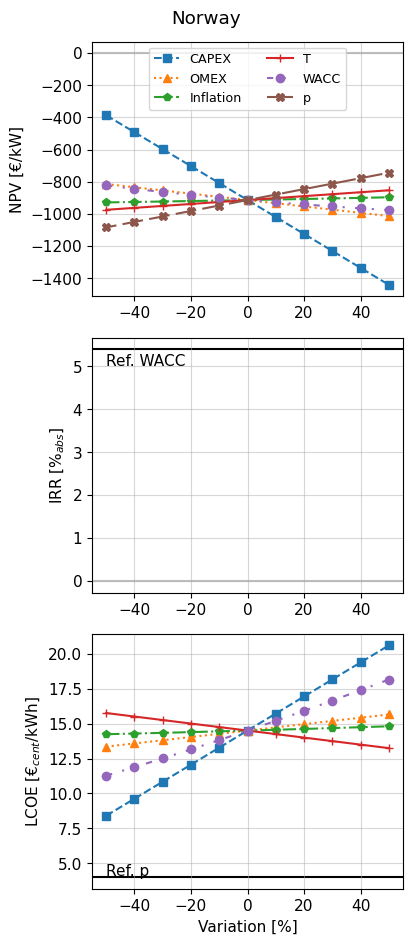

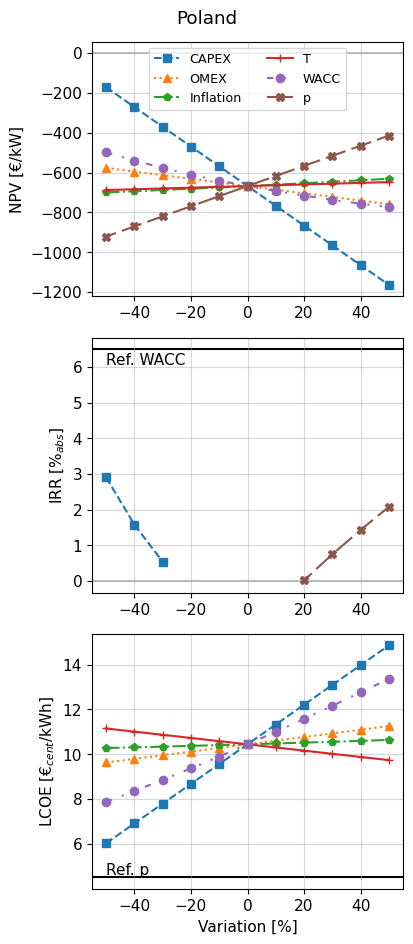

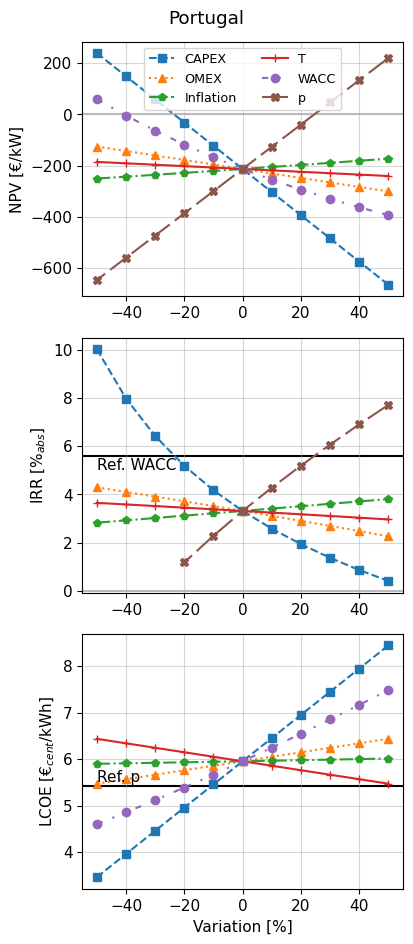

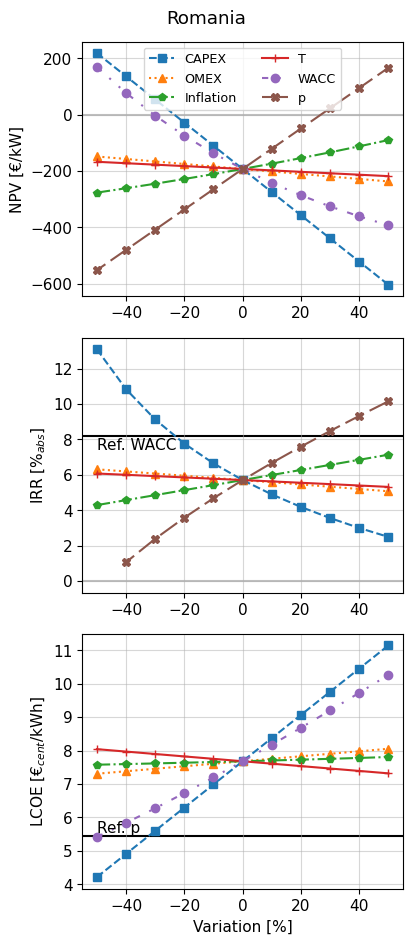

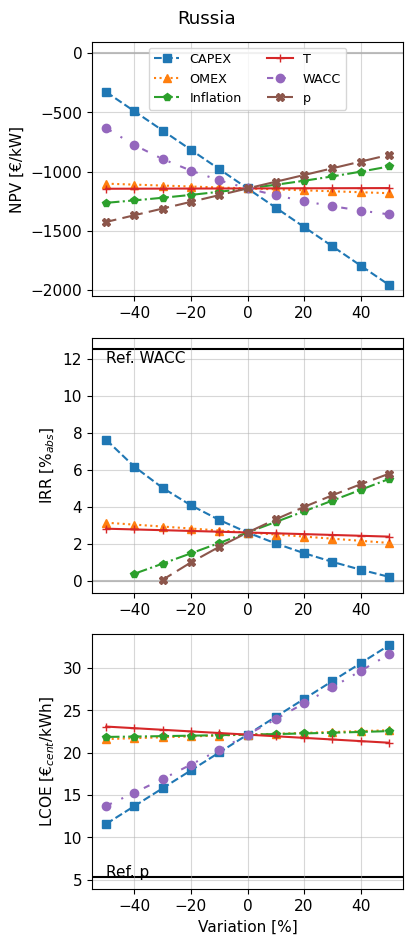

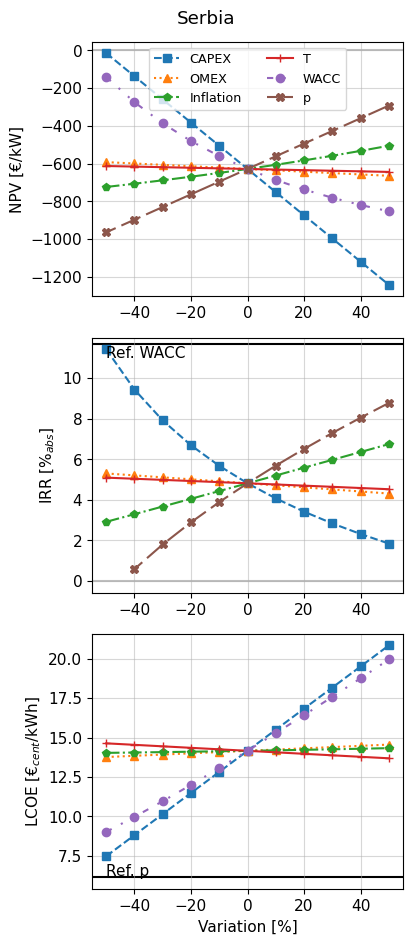

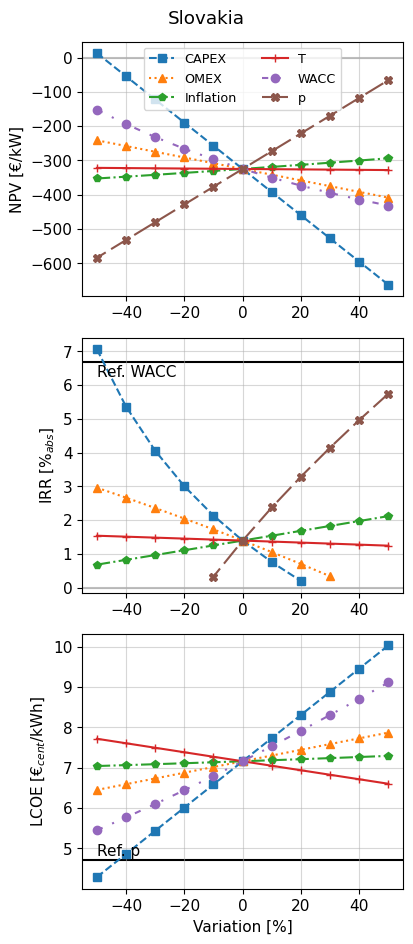

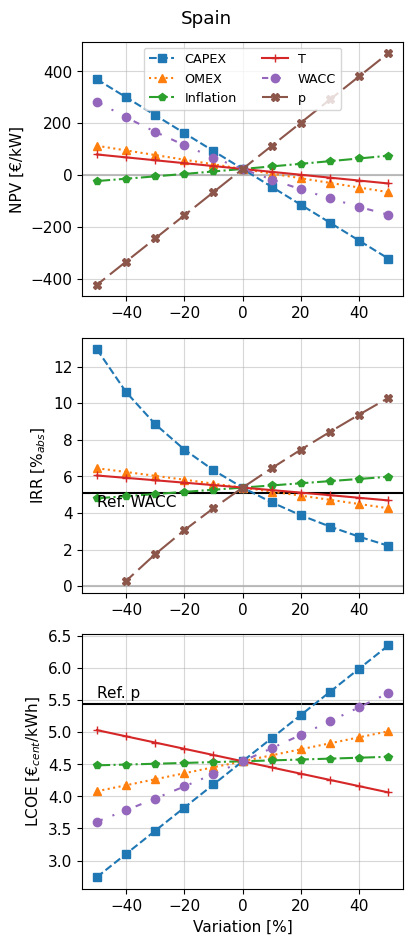

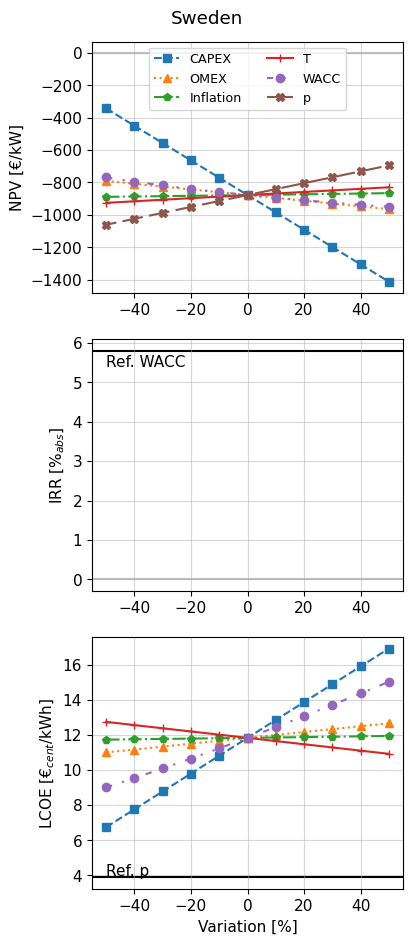

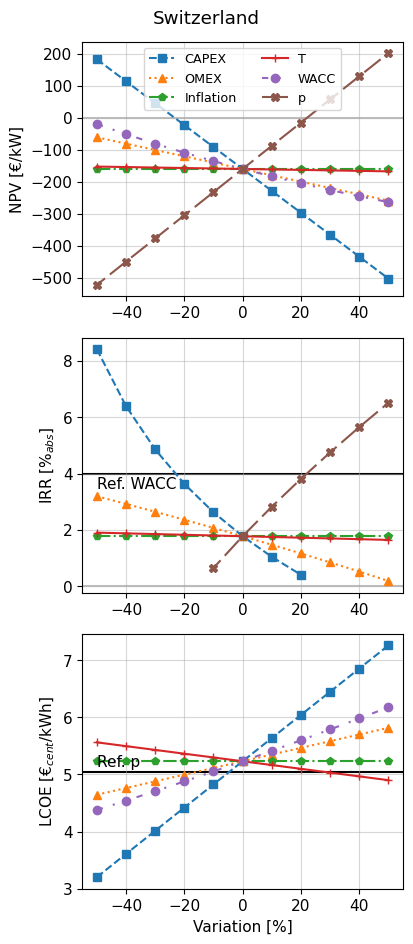

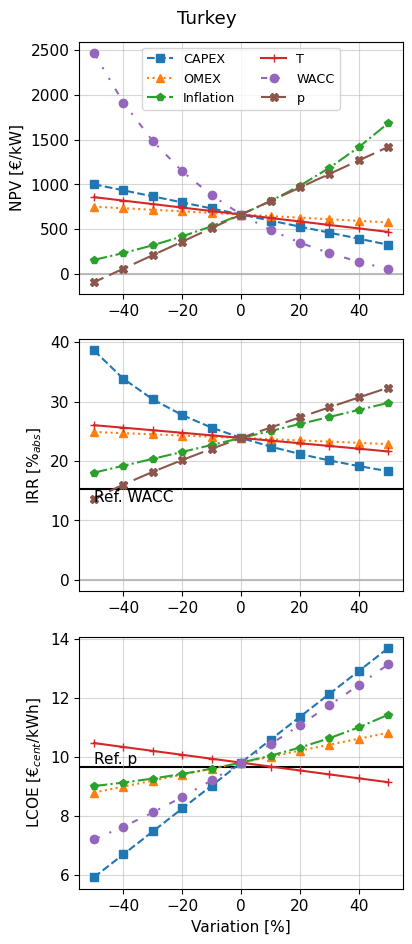

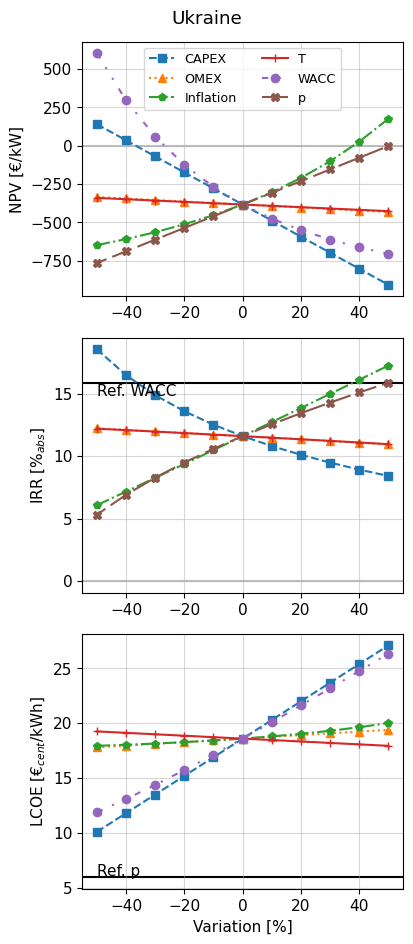

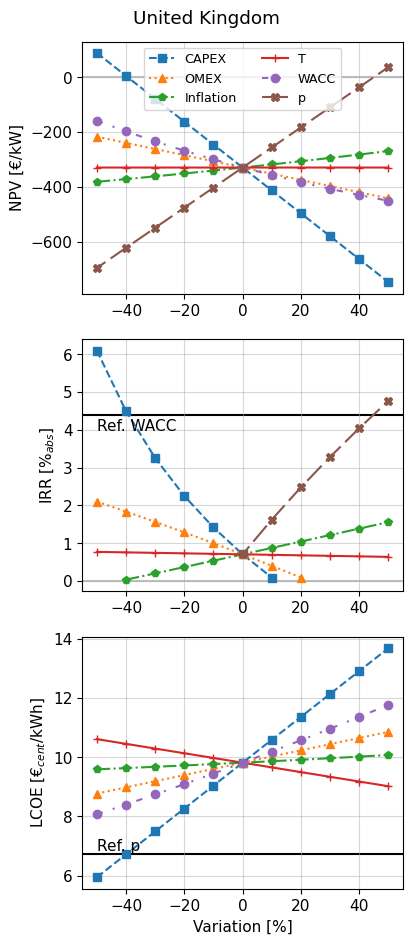
**

Supplement: Multimedia component 1 [file mmc1.docx]
